# The Role of the Stem Cells Therapy in the Peripheral Artery Disease

**DOI:** 10.3390/ijms20092233

**Published:** 2019-05-07

**Authors:** Federico Biscetti, Nicola Bonadia, Elisabetta Nardella, Andrea Leonardo Cecchini, Raffaele Landolfi, Andrea Flex

**Affiliations:** 1 Fondazione Policlinico Universitario A. Gemelli IRCCS, U.O.C. Clinica Medica e Malattie Vascolari, 00168 Roma, Italy; elisabetta.nardella@gmail.com (E.N.); andrealeonardo-@hotmail.it (A.L.C.); raffaele.landolfi@unicatt.it (R.L.); andrea.flex@unicatt.it (A.F.); 2 Laboratory of Vascular Biology and Genetics, Università Cattolica del Sacro Cuore, 00168 Roma, Italy; nicola.bonadia@gmail.com; 3 Fondazione Policlinico Universitario A. Gemelli IRCCS, U.O.C. Medicina d’Urgenza e Pronto Soccorso, 00168 Roma, Italy; 4 Università Cattolica del Sacro Cuore, 00168 Roma, Italy

**Keywords:** Stem cells therapy, type 2 diabetes mellitus, lower limb peripheral artery disease

## Abstract

Vascular complications of diabetes mellitus are an important issue for all clinicians involved in the management of this complex pathology. Although many therapeutic advances have been reached, peripheral arterial disease is still an unsolved problem that each year compromises the quality of life and life span of affected patients. Oftentimes, patients, after ineffective attempts of revascularization, undergo greater amputations. At the moment, there is no effective and definitive treatment available. In this scenario, the therapeutic use of stem cells could be an interesting option. The aim of the present review is to gather all the best available evidence in this regard and to define a new role of the stem cells therapy in this field, from biomarker to possible therapeutic target.

## 1. Background

Atherosclerosis is the leading cause of death worldwide, with myocardial infarction and stroke accounting for almost 15 million deaths in 2015 [1]. Lower limb peripheral artery disease (LL-PAD) belongs to the pathological spectrum of atherosclerosis and is an important cause of death and disability, with a prevalence ranging between 3% and 10% in the general population and as high as 15% to 20% in persons aged >70 years [2,3,4]. This population has an increased risk of cardiovascular and cerebrovascular accidents, and up to 10% of them will progress to critical limb ischemia (CLI), which is the final stage of LL-PAD. Such figure corresponds to and incidence of 500–1000 per million in the developed world. As prevalence of diabetes mellitus increases, especially in developing countries, and with the ageing of the population, these figures are likely to rise in the foreseeable future. Patients with CLI, in turn, have a reduced life expectancy and a significant morbidity, due to recurrent soft tissue infections, difficult-to-treat ischemic pain, reduced mobility and limb amputation. Despite considerable improvement in the pharmacological therapy and the availability of endovascular and surgical treatment for CLI, about 50% of patients with CLI undergoing treatment will be dead or amputated at a one-year follow-up [5]. Thus, while effective, there is still an unmet therapeutic need in the treatment of patients with critical limb ischemia. In the last two decades, therapeutic angiogenesis has emerged as a new possibility in the management of CLI. Therapeutic angiogenesis refers to the use of directly inoculated angiogenetic factors, genes or cells in the ischemic limb in order to induce or improve the physiological process of collateral vessel formation. While tempting, this option is still unavailable for routine clinical use. The aim of this review is to offer an overview of cell-based therapeutic angiogenesis, to summarize the results of the main clinical trials in this field, to highlight the gap in the evidence that prevents this strategy from being routinely used.

## 2. Overview of Stem Cells

Almost all stem cells can be classified according to their degree of potency and the related site of harvesting. Thus, each stem cell population belongs to one of four classes:

- Totipotent stem cells, which are able, in principle, to give rise to a complete individual; embryonal cells, up to the four-blastomeres stage, belong to this group [6,7];
- Pluripotent stem cells, which are able to differentiate into the three embryonal sheaths but not into extra-embryonal structure; embryonal stem cells belong to this group [8];
- Multipotent stem cells, which are able to differentiate into a restricted number of cell types; adult (also called somatic) stem cells, which are cells whose role is the renewal of self-renewing tissues, belong to this group;
- Unipotent stem cells, which are able to differentiate into a single cell type; for example, lineage-committed hematopoietic cells belong to this type.

Totipotent and pluripotent stem cells are, potentially, the ideal source for tissue engineering. However, ethical and clinical concerns limit their use and, while an area of active research, they will probably not be available at the bedside in the near future. Conversely, multipotent stem cells are widely available from multiple adult tissues and, unlike unipotent stem cells, have sufficient plasticity for tissue repairing. Multipotent stem cells harvested from bone marrow and from adipose tissue have actually been tested for therapeutic angiogenesis in multiple clinical trials (Table S1).

Asahara et al. first demonstrated that hematopoietic stem cells (HSC) can differentiate into endothelial cells and can sustain angiogenesis in adult individuals [9]. Bone marrow contains also a population of non-hematopoietic stem cells, which present a spindle-shaped structure and are able to give rise to mature mesenchymal tissues (cartilage, bone, adipose tissue, connective tissue) in culture conditions, defined mesenchymal stem cells [10]. Mesenchymal stem cells (MSCs) have been isolated from multiple adult tissues, including bone marrow and adipose tissue [11,12,13,14], and are thought of as a cell population with interesting characteristics, including an immunoprivileged status, the ability to secrete paracrine factors with angiogenetic, immunomodulatory and anti-inflammatory properties [15,16]. They have been used in treating graft-versus-host disease [17] and are a promising tool for autoimmune disease. Moreover, they seem to be able to both differentiate into endothelial cells and to promote angiogenesis indirectly via two mechanisms: paracrine stimulation of endothelial progenitor cells and direct participation in the formation of the mesenchymal scaffold required for effective vessel formation [18,19,20,21,22]. Currently, there is not a single surface marker which can be used for mesenchymal cell identification and such cells have been traditionally been defined by their in vitro properties (spindle shape, plastic-adherence, ability to differentiate into chondroblasts, osteoblasts and adipocytes). In 2006, the International Society of Stem Cell Therapy has issued a minimum set of criteria for mesenchymal stem cell definition: apart from the said characteristics, they must not express hematopoietic or endothelial commitment markers (CD45, CD34, CD14 or CD11b, CD79alpha or CD19 and HLA-DR) and they must express CD73, CD105 and CD90 [23].

**Figure 1:**
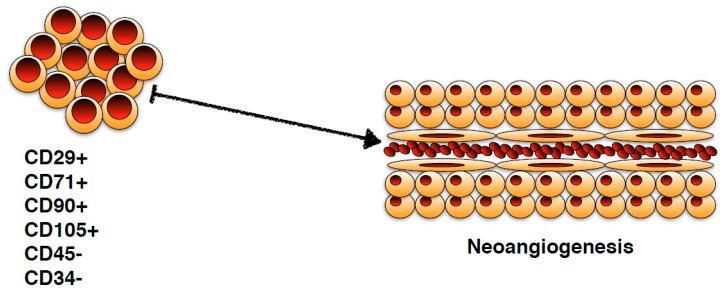
A schematic representation of bone marrow stem cells function. Starting from different immunophenotypes, these cellular populations are able to form new vascular tissue, consisting of endothelial cells and extravascular structures.

Given the above considerations, the availability and relative ease of collection, bone marrow stem cells have been the most investigated type of stem cells for therapeutic angiogenesis (Figure 1). To understand clinical trials on this topic, one must keep in mind the characteristics that distinguish each trial, which can be summarized as follows:

1. Specific stem cell population: some trials have used hematopoietic stem cells (based on the assumption that these cells differentiate into endothelial cells), bone-marrow-derived mesenchymal stem cells (based on the fact that these cells are able to differentiate, in vitro, into endothelial cells and are able to give rise to pericytes, which are the required scaffold for newly-formed vessels and, moreover, are able to secrete paracrine factors that form the intracellular milieu for angiogenesis) or a mixed population;
2. Harvesting mode: most trials have used direct bone-marrow aspiration, while a limited number of trials have used circulating HSCs, after mobilization by colony-stimulating factors;
3. Possible in vitro expansion of harvested cells (especially for MSCs);
4. Exact immunophenotypical characterization of cells;
5. Phase (most studies are phase 1-2 studies, while only a single published study is a phase 3 trial).

Besides their direct role in angiogenesis, stem cells may be used as gene therapy vectors. Given its pivotal role in post-natal angiogenesis and in tumoral angiogenesis [24], vascular endothelial growth factor (VEGF) administration has gained a lot of interest as a potential angiogenetic therapy in different ischemic states [25], with partially disappointing results [26,27,28,29,30,31,32,33]. The lack of efficacy of VEGF therapy is probably linked to the fact that normal or aberrant vascular growth is strictly dependent in microenvironmental VEGF concentration and on sufficient duration of VEGF stimulation to spurring vessels; thus, it cannot be adequately controlled by systemic administration of either the protein itself or VEGF gene-containing plasmids [34,35,36,37,38,39]. Stem cells may be a key to overcome this limitation. For example, VEGF expression has been induced in myoblasts [40] and, more recently, myoblasts populations with a uniform and known expression of VEGF have been isolated and shown to induce angiogenesis without giving rise to aberrant vascular structure in nude mice [41]. This approach has also been applied to MSCs. For example, Fierro et al. induced expression of platelet derived growth factor subunit B (PDGF-B), VEGF, basic fibroblast growth factor (bFGF), transforming growth factor-β (TGF-β) in marrow-derived MSCs, showing that different induced expression of the aforementioned factors could alter the fate of MSCs after implantation or their effect on angiogenesis [42]. They also showed that such a population could be effective and safe in an animal model [43] and could be efficiently produced according to Good Manufacturing Practices [44]. Stable and uniform VEGF expression has been obtained also in adipose-derived mesenchymal stem cells (ASC), which proved effective in slowing decline in ejection fraction of murine heart after myocardial infarction [45,46].Further combination are possible by combining gene transfection with systemic, local or pre-injection treatment with specific soluble factors [47].

As MSCs tend to home in sites of hypoxic injury, can differentiate into pericytes and secrete several growth factors, gene-transfer-enhanced MSCs could be the missing link between gene- and cell-based therapies. However, such cells have been generated by retroviral or lentiviral vectors. Thus, immunogenicity of the final product as well as the risk of potential insertional mutagenesis remain concerning. Plasmid gene delivery is less efficient than viral gene delivery but, in principle, safer. Moreover, plasmids may be lost during in vivo replication of injected cells. To address the first point, co-expression of the desired gene with a marker gene could be used to select the actually transfected population out of a pool of cells which have undergone a plasmid transfection procedure. Park et al. have shown that co-expression of VEGF with the enhanced green fluorescent protein (EGFP) allowed the enrichment of a starting population of MSCs via flow cytometry. Such enriched population has proved effective in the animal model at 21 days [48]. It is worth noticing that Park et al. [48] point out that the ability of MSCs to create an angiogenetic environment makes them the ideal candidate for combined cell/gene therapy in ischemic disease.

As PAD is a particularly common in diabetic patients, one question is whether impaired metabolic pathways lead to a decreased angiogenetic capacity of diabetic patients’ stem cells. In fact, together with accelerated atherosclerosis, impaired angiogenesis has been called into question to explain the increased susceptibility of this population to vascular disease [49,50,51,52,53,54]. Such impaired angiogenesis seems, at least in part, to be linked to reduced response to VEGF by circulating endothelial progenitor cells [55,56,57], to reduced absolute circulating number of endothelial progenitor cells and to stem cells dysfunction [58,59,60,61]. Diabetes mellitus may, furthermore, be deleterious also for MSCs [62]. Based on these premises, it has been postulated that pretreatment with specific signaling proteins could alter the functionality of stem cells to induce a more clinically efficient phenotype. Thus, Amin et al. [63] stimulated BM-derived MSCs from diabetic mice with epidermal growth factor before injection into ischemic hindlimbs. Pre-treatment with EGF resulted in accelerated angiogenesis compared with diabetic mice who received non-pretreated MSCs and this effect was probably due to increased expression of phosphorylated VEGF-R and Akt in the former. In vitro, they observed and increased adhesion and migration of pre-treated cells compared to non-pretreated cells. In keeping with these results, our group has shown that HMBG-1, albeit at supraphysiological concentration, is capable of inducing vascular differentiation of stem cells and that this effect is dependent on the VEGF pathway [64], while another group has shown that IL-8 may be another potential candidate to enhance angiogenetic capabilities of stem cells [65]. Given their immunomodulatory properties, allogenic MSCs may be an alternative both to overcome the diabetes-induced dysfunction of autologous MSCs and to allow for more economically efficient source of MSCs [66,67].

## 3. Human Trials of Stem Cells Therapy

In 2002, Tateishi-Yuyama et al. [68] performed a landmark trial on the use of a mixed population of bone-marrow-derived CD34+ and CD34- cells for no-option critical limb ischemia. In their study, they performed both a pilot phase and a subsequent study with formal sample size assessment. Cells did not undergo an in-vitro expansion phase and were only sorted and concentrated before limb implantation. They found a marginal increase in ankle/brachial index (ABI) values in treated limbs compared with untreated limbs (+0.1). However, they found a noteworthy increase in TcPO2 (+12.00 mm Hg compared with saline-treated limbs, with an overall absolute increase of 16.6 mm Hg compared to baseline). 16 out of 20 patients in the treatment group experienced complete resolution of rest pain, versus only 3 out of 20 in the control group. Magnetic resonance angiography (MRA) showed an increase in collateral vessels in the treatment group compared with the control group, consistent with the clinical improvement. A major strength of this study is the blinding of all investigators prior to treatment assignment of patients. After randomization, authors report that blinding was maintained for all the involved personnel, except for each center principal investigator. Obviously, a complete blinding up to and including outcome assessment for all involved personnel, including principal investigators, would have made results more reliable. Moreover, there was a “limb-level” randomization, which entailed that in each patient, a single limb was used as a treatment limb while the other served as control. While this approach made the two groups more comparable in term of baseline characteristics, it prevented the study to be able to assess possible systemic adverse events or benefits. The small increase in ABI with a proportionally bigger increase in TcPO2 may be explained by the fact that neo-angiogenesis is expected to occur at the smaller vessel level. That is, large vessel patency is unlikely to be affected by stem cells implantation, thus explaining small increases in ABI values, while small vessel collaterals may support distal limb oxygenation even without a significant increase in blood pressure. Moreover, in control group limb peripheral blood mononuclear cells were implanted, which may have exerted some potentially beneficial effect, thus mitigating the observed effect of stem cells therapy. It should also be noted that patients enrolled in this study had an ABI below 0.6, thus representing a particularly advanced form of PAD. Aside from clinical findings, authors also performed molecular analysis on the used cells. It is noteworthy that CD34− cells (i.e., cells that were not hematopoietic progenitors, HSC) had the highest expression of mRNAs codifying for bFGF, angiopoietin-1 and VEGF, whereas CD34+ cells showed more mRNAs codifying for the respective receptors, thus supporting the concept that marrow-derived MSCs are necessary to support HSCs angiogenesis.

Those encouraging results were partly replicated in 2005 by Huang et al. [69]. These authors focused exclusively on diabetic patients, both type 1 and type 2, and used peripheral blood mononuclear cells after mobilization via administration of granulocyte colony stimulating factor (G-CSF). While they obtained an impressive improvement in treated patients versus control patients, they did not report about randomization procedure, and the study was performed open label. Moreover, treated group both received G-CSF and intravenous heparin, while the control group received prostaglandin E1 infusion, thus raising the possibility that the effect seen may be partly attributable to those differential treatment. Additionally, they did not perform a formal sample size calculation and did not specify the statistical power of the study. It is worth noticing that these authors reported improvement in glycemic control in treated subjects. They assume that this effect may be due to an action of stem cells on pancreatic β-cells; however, taking into account the above consideration, this may also be an indirect evidence of performance bias.

Both those studies used mixed bone-marrow cells populations. Thus, they were not designed to evaluate whether mesenchymal stem cells or mixed mononuclear cells were responsible for the reported clinical benefit. In 2011, Lu et al. [70] performed a three-arms study to both evaluate whether stem cells therapy is effective in CLI and to evaluate the relative benefit of mixed bone marrow population and mesenchymal stem cells. In their study, they separately compared both a mixed population of bone-marrow derived mononuclear cells (BM-MNCs) and sorted bone-marrow mesenchymal stem cells (BM-MSCs) with a placebo group of limbs, in which only normal saline was injected. BM-MSCs were sorted via Ficoll gradient centrifugation from a 30 mL marrow aspirate and then expanded in vitro before injection, while BM-MNCs did not undergo expansion. They showed clinical benefit upon control for both the treatments, with a more marked increase for limbs receiving mesenchymal stem cells. This benefit included a 100% ulcer healing and no amputation in the treated limbs. This study represents a landmark trial in cell therapy for several reasons. First, this is the first study to directly compare mononuclear cells and mesenchymal stem cells; secondly, the MSCs underwent immunophenotypical testing and complied with the IFSCT criteria for mesenchymal stem cells definition; third, this study showed that in vitro expanded MSCs did not induce adverse event at a 24-week follow-up. Moreover, this study showed that MSCs treatment resulted in greater improvement in clinical outcomes than mixed mononuclear cells. Aside from clinical outcomes, the authors also showed that MSCs produce a greater amount of angiopoietic factors (specifically VEGF, angiopoietin-1 and bFGF) than MNCs and this difference is increased in hypoxic conditions. As hematopoietic stem cells (CD34+) are known to physiologically circulate and take part in angiogenesis, this study may support the idea that the bottleneck in angiogenesis in CLI patients is represented by the availability of MSCs. Thus, the higher number of MSCs injected in BM-MSCs treated patients may explain the greater improvement in these subjects. From a methodological point of view, this study is reported to be randomized, double-blind and single center. While randomization has been conducted with two independent randomization tables (one to assign patients to MSC or MNC group and the other to assign each limb of each patient to treatment or to control group), how blinding was attained and maintained has not been reported. Moreover, MSCs treatment required the aspiration of a far smaller amount of marrow blood compared to MNCs treatment (30 vs. 300 mL). However, the in vitro expansion procedure is time-consuming and may represent a limit in routine implementation of their technique.

In 2012, Ozturk et al. [71] replicated the results by Huang et al. [69]. They enrolled 20 treatment patients to be injected with GM-CSF mobilized peripheral blood CD34+ cells and 20 control patients, showing a small but meaningful difference in clinical parameters after 12 weeks of follow-up. Of note, they did not retransplant patients after 40 days if required (like in the Huang’s paper). However, the small sample size, the single center and open label nature of the study make their result insufficient for clinical use. Moreover, the management of control group, apart for best medical therapy, is unclear. Of note, control patients, unlike treated patients, did not receive GM-CSF or heparin.

While other smaller studies have confirmed these results, they are all pilot studies, with small sample size or inadequate blinding [72,73]. However, In the same year, the RESTORE-CLI, the first multicenter and sponsor-initiated study has been published [74,75]. In this study, a cellular product named Ixymielocel-T, crafted from each patient’s bone marrow stem cells, has been used. Ixymielocel-T was a mixed population of MSCs and HSCs (CD90+ the former, CD34+ the latter) which underwent expansion by a proprietary procedure. As reported, only MSCs (i.e. the CD90+ fraction of bone-marrow aspirate) and macrophage-committed HSCs (CD14+) underwent in vitro expansion. Thus, Ixymielocel-T may be thought of as a variant of in vitro expanded MSCs (given that expanded CD90+ cells were the major constituent of the final product). This was a phase 2 study and, as such, was designed to evaluate safety and was not powered to evaluate efficacy over placebo. However, this is the first multicenter study, with 18 centers across USA and with reliable blinding procedures reported. It enrolled CLI patients who were not amenable to surgical or endovascular revascularization procedures and, apart from safety, showed efficacy of the cell therapy on a composite endpoint of treatment failures (defined as the time to first occurrence of a treatment failure, which, in turn, consisted of the composite of major amputation, doubling of the ulcers area, de novo gangrene and all-cause mortality). The study did not reach statistical significance on the endpoint of amputation-free survival. Again, the RESTORE-CLI was a phase 2 trial, which had as strengths, its multicenter, randomized and double blinded nature and which gave encouraging results. It is worth noticing that Ixymielocel-T showed benefits also in patients with cardiac failure [76,77]. The RESTORE-CLI and the above-mentioned TACT studies were the only ones to enroll only CLI patients without surgical options.

Subsequently, there has been an increased interest on the use of MSCs in CLI. Two studies evaluated the efficacy of MSCs therapy in CLI. The first study [78], while performed with the declared intent of using exclusively MSCs, specifically peripheral blood MSCs after mobilization with G-CSF, critically, did not specify how MSCs were sorted nor any immunophenotyping. However, it showed impressive results (with no amputation in the treatment group after three months, versus a 50% amputation rate in the control group). The second study was a phase 1 trial [79] which, for the first time, used allogenic stem cells. Investigators performed immunophenotyping on the stem cells, so to satisfy ISCT criteria for MSCs and did not use immunosuppressive drugs (notwithstanding the fact that donors were not HLA-matched to recipients), thus leveraging the immunomodulatory properties of MSCs. This study was also a multicenter study, with central randomization. Being a phase 1 trial, there was no blinding. The authors reported, again, impressive results with respect to ABI and rest pain, but with no significant difference in amputation rates. Again, the small sample size and the phase 1 nature of this study may have prevented it from showing statistically meaningful differences on this clinical endpoint. However, the use of allogenic MSCs without the need for HLA matching nor immunosuppressive therapy is intriguing and may pave the way to off-the-shelf MSCs products which may be able to overcome the limitation of the long time required for patient-specific preparations.

Another interesting development of stem cells therapy is the possibility to use MSCs not derived from bone marrow. As said above, MSCs are found in many different tissue types and they are particularly abundant in adipose tissue. Thus, adipose tissue may represent a source of abundant, readily available stem cells, without the need for bone marrow aspiration. Joining this concept with that of allogenic stem cells transplantation may pave the way for liposuction-derived off-the-shelf cellular products, without the need of bone marrow donation or the need for patient-specific preparation. The use of autologous adipose tissue-derived MSCs has been tested in a phase 1 trial [80] which showed overall safety of this approach, with a putative benefit (seen only on wound size). It is worth noticing that meta-analysis on the use of stem cell therapy in CLI have confirmed the benefits observed in single studies [81,82], but pointing out methodological issues which limit their applicability to clinical practice [83].

## 4. Conclusions

The therapeutic use of stem cells represents an interesting new direction in the management of vascular complications of diabetes mellitus and, in general, of peripheral artery disease. In our opinion, several issues should be addressed before cell therapy may be part of routine clinical practice. First, while none of the studies reviewed in our article showed potential adverse effect of stem cell therapy, it is clear that none of these studies was large enough to detect potentially rare adverse event. Moreover, very long follow-up would be needed to exclude the possibility of tumorigenesis by implanted stem cells. However, it is worth noticing that no such potential concern has been raised by any of the reviewed studies. Second, it is not clear whether administration of stem cells at an earlier stage of disease could be more beneficial. All the studies reviewed in this paper have enrolled patients with clinically evident CLI. Furthermore, CLI is known to be the end stage of lower limb atherosclerosis. Thus, once longer follow-up on CLI patients will be available, there is need of further research to clarify whether earlier use of stem cell therapy can affect the course of disease.

We also focused on the possible pre-implantation treatment of stem cells with various growth factors to improve their angiogenetic properties. In principle, a better understanding of the atherosclerotic process could lead to personalized treatment for different group of patients. For example, the use of specific injected genes or different growth factors for pre-treatment of stem cells could be based on specific phenotypical characteristics of the patient, such as the presence or not of diabetes, a history of smoking or specific circulating markers.

Thus far, however, the most important unmet need in the field of cell therapy for PAD is the lack of methodologically sound phase 3 randomized controlled trials to ultimately show clinically significant benefit of stem cell therapy.

Although many points are still unclear and additional data are needed, it is important that this field be explored with following studies to design a new therapeutic approach available in this scenario.

## Supplementary Materials

Supplementary materials can be found at https://www.mdpi.com/1422-0067/20/9/2233/s1.

## References

[1] GBD 2015 Mortality and Causes of Death Collaborators (2016). Global, regional, and national life expectancy, all-cause mortality, and cause-specific mortality for 249 causes of death, 1980–2015: A systematic analysis for the Global Burden of Disease Study 2015. Lancet.

[2] Criqui M.H., Fronek A., Barrett-Connor E., Klauber M.R., Gabriel S., Goodman D. (1985). The prevalence of peripheral arterial disease in a defined population. Circulation.

[3] Hiatt W.R., Hoag S., Hamman R.F. (1995). Effect of diagnostic criteria on the prevalence of peripheral arterial disease. The San Luis Valley Diabetes Study. Circulation.

[4] Selvin E., Erlinger T.P. (2004). Prevalence of and risk factors for peripheral arterial disease in the United States: Results from the National Health and Nutrition Examination Survey, 1999–2000. Circulation.

[5] Norgren L., Hiatt W.R., Dormandy J.A., Nehler M.R., Harris K.A., Fowkes F.G.R. (2007). Inter-Society Consensus for the Management of Peripheral Arterial Disease (TASC II). Eur. J. Vasc. Endovasc. Surg..

[6] Mitalipov S.M., Yeoman R.R., Kuo H.-C., Wolf D.P. (2002). Monozygotic twinning in rhesus monkeys by manipulation of in vitro-derived embryos. Biol. Reprod..

[7] Tarkowski A.K. (1959). Experiments on the development of isolated blastomers of mouse eggs. Nature.

[8] Thomson J.A., Itskovitz-Eldor J., Shapiro S.S., Waknitz M.A., Swiergiel J.J., Marshall V.S., Jones J.M. (1998). Embryonic stem cell lines derived from human blastocysts. Science.

[9] Asahara T., Murohara T., Sullivan A., Silver M., van der Zee R., Li T., Witzenbichler B., Schatteman G., Isner J.M. (1997). Isolation of putative progenitor endothelial cells for angiogenesis. Science.

[10] Castro-Malaspina H., Gay R.E., Resnick G., Kapoor N., Meyers P., Chiarieri D., Mckenzie S., Broxmeyer H.E., Moore M.A. (1980). Characterization of human bone marrow fibroblast colony-forming cells (CFU-F) and their progeny. Blood.

[11] Da Silva Meirelles L., Chagastelles P.C., Nardi N.B. (2006). Mesenchymal stem cells reside in virtually all post-natal organs and tissues. J. Cell Sci..

[12] Fraser J.K., Wulur I., Alfonso Z., Hedrick M.H. (2006). Fat tissue: An underappreciated source of stem cells for biotechnology. Trends Biotechnol..

[13] Aust L., Devlin B., Foster S.J., Halvorsen Y.D.C., Hicok K., Laney T.D., Sen A., Willingmyre G.D., Gimble J.M. (2004). Yield of human adipose-derived adult stem cells from liposuction aspirates. Cytotherapy.

[14] Zuk P.A., Zhu M., Ashjian P., De Ugarte D.A., Huang J.I., Mizuno H., Alfonso Z.C., Fraser J.K., Benhaim P., Hedrick M.H. (2002). Human adipose tissue is a source of multipotent stem cells. Mol. Biol. Cell.

[15] Urban V.S., Kiss J., Kovacs J., Gocza E., Vas V., Monostori Ė., Uher F. (2008). Mesenchymal Stem Cells Cooperate with Bone Marrow Cells in Therapy of Diabetes. Stem Cells.

[16] Aggarwal S. (2005). Human mesenchymal stem cells modulate allogeneic immune cell responses. Blood.

[17] Le Blanc K., Frassoni F., Ball L., Locatelli F., Roelofs H., Lewis I., Lanino E., Sundberg B., Bernardo M.E., Remberger M. (2008). Mesenchymal stem cells for treatment of steroid-resistant, severe, acute graft-versus-host disease: A phase II study. Lancet.

[18] Oswald J., Boxberger S., Jørgensen B., Feldmann S., Ehninger G., Bornhäuser M., Werner C. (2004). Mesenchymal stem cells can be differentiated into endothelial cells in vitro. Stem Cells.

[19] Hausman G.J., Richardson R.L. (2004). Adipose tissue angiogenesis. J. Anim. Sci..

[20] Rehman J., Traktuev D., Li J., Merfeld-Clauss S., Temm-Grove C.J., Bovenkerk J.E., Pell C.L., Johnstone B.H., Considine R.V., March K.L. (2004). Secretion of angiogenic and antiapoptotic factors by human adipose stromal cells. Circulation.

[21] Planat-Benard V., Silvestre J.S., Cousin B., André M., Nibbelink M., Tamarat R., Clergue M., Manneville C., Saillan-Barreau C., Duriez M. (2004). Plasticity of human adipose lineage cells toward endothelial cells: Physiological and therapeutic perspectives. Circulation.

[22] Miranville A., Heeschen C., Sengenès C., Curat C.A., Busse R., Bouloumié A. (2004). Improvement of postnatal neovascularization by human adipose tissue-derived stem cells. Circulation.

[23] Dominici M.L.B.K., Le Blanc K., Mueller I., Slaper-Cortenbach I., Marini F.C., Krause D.S., Deans R.J., Keating A., Prockop D.J., Horwitz E.M. (2006). Minimal criteria for defining multipotent mesenchymal stromal cells. The International Society for Cellular Therapy position statement. Cytotherapy.

[24] Carmeliet P. (2003). Angiogenesis in health and disease. Nat. Med..

[25] Matsumoto K., Ema M. (2014). Roles of VEGF-A signalling in development, regeneration, and tumours. J. Biochem..

[26] Fortuin F.D., Vale P., Losordo D.W., Symes J., DeLaria G.A., Tyner J.J., Schaer G.L., March R., Snell R.J., Henry T.D. (2003). One-year follow-up of direct myocardial gene transfer of vascular endothelial growth factor-2 using naked plasmid deoxyribonucleic acid by way of thoracotomy in no-option patients. Am. J. Cardiol..

[27] Rajagopalan S., Mohler E.R., Lederman R.J., Mendelsohn F.O., Saucedo J.F., Goldman C.K., Blebea J., Macko J., Kessler P.D., Rasmussen H.S. (2003). Regional angiogenesis with vascular endothelial growth factor in peripheral arterial disease: A phase II randomized, double-blind, controlled study of adenoviral delivery of vascular endothelial growth factor 121 in patients with disabling intermittent claudication. Circulation.

[28] Rajagopalan S., Mohler E., Lederman R.J., Saucedo J., Mendelsohn F.O., Olin J., Blebea J., Goldman C., Trachtenberg J.D., Pressler M. (2003). Regional Angiogenesis with Vascular Endothelial Growth Factor (VEGF) in peripheral arterial disease: Design of the RAVE trial. Am. Heart J..

[29] Deev R., Plaksa I., Bozo I., Isaev A. (2017). Results of an International Postmarketing Surveillance Study of pl-VEGF165 Safety and Efficacy in 210 Patients with Peripheral Arterial Disease. Am. J. Cardiovasc. Drugs.

[30] Deev R.V., Bozo I.Y., Mzhavanadze N.D., Voronov D.A., Gavrilenko A.V., Chervyakov Y.V., Staroverov I.N., Kalinin R.E., Shvalb P.G., Isaev A.A. (2015). pCMV-vegf165 Intramuscular Gene Transfer is an Effective Method of Treatment for Patients with Chronic Lower Limb Ischemia. J. Cardiovasc. Pharmacol. Ther..

[31] Losordo D.W., Vale P.R., Hendel R.C., Milliken C.E., Fortuin F.D., Cummings N., Schatz R.A., Asahara T., Isner J.M., Kuntz R.E. (2002). Phase 1/2 placebo-controlled, double-blind, dose-escalating trial of myocardial vascular endothelial growth factor 2 gene transfer by catheter delivery in patients with chronic myocardial ischemia. Circulation.

[32] Bashir R., Vale P.R., Isner J.M., Losordo D.W. (2002). Angiogenic gene therapy: Pre-clinical studies and phase I clinical data. Kidney Int..

[33] Stewart D.J., Kutryk M.J., Fitchett D., Freeman M., Camack N., Su Y., Della Siega A., Bilodeau L., Burton J.R., Proulx G. (2009). VEGF gene therapy fails to improve perfusion of ischemic myocardium in patients with advanced coronary disease: Results of the NORTHERN trial. Mol. Ther..

[34] Ozawa C.R., Banfi A., Glazer N.L., Thurston G., Springer M.L., Kraft P.E., McDonald D.M., Blau H.M. (2004). Microenvironmental VEGF concentration, not total dose, determines a threshold between normal and aberrant angiogenesis. J. Clin. Investig..

[35] von Degenfeld G., Banfi A., Springer M.L., Wagner R.A., Jacobi J., Ozawa C.R., Merchant M.J., Cooke J.P., Blau H.M., von Degenfeld G. (2006). Microenvironmental VEGF distribution is critical for stable and functional vessel growth in ischemia. FASEB J..

[36] Park J.E., Keller G.A., Ferrara N. (1993). The vascular endothelial growth factor (VEGF) isoforms: Differential deposition into the subepithelial extracellular matrix and bioactivity of extracellular matrix-bound VEGF. Mol. Biol. Cell.

[37] Springer M.L., Chen A.S., Kraft P.E., Bednarski M., Blau H.M. (1998). VEGF gene delivery to muscle: Potential role for vasculogenesis in adults. Mol. Cell.

[38] Lander A.D. (2007). Morpheus unbound: Reimagining the morphogen gradient. Cell.

[39] Banfi A., von Degenfeld G., Blau H.M. (2005). Critical role of microenvironmental factors in angiogenesis. Curr. Atheroscler. Rep..

[40] Banfi A., Springer M.L., Blau H.M. (2002). Myoblast-mediated gene transfer for therapeutic angiogenesis. Methods Enzymol..

[41] Misteli H., Wolff T., Füglistaler P., Gianni-Barrera R., Gürke L., Heberer M., Banfi A. (2010). High-throughput flow cytometry purification of transduced progenitors expressing defined levels of vascular endothelial growth factor induces controlled angiogenesis in vivo. Stem Cells.

[42] Fierro F.A., Kalomoiris S., Sondergaard C.S., Nolta J.A. (2011). Effects on proliferation and differentiation of multipotent bone marrow stromal cells engineered to express growth factors for combined cell and gene therapy. Stem Cells.

[43] Beegle J.R., Magner N.L., Kalomoiris S., Harding A., Zhou P., Nacey C., White J.L., Pepper K., Gruenloh W., Annett G. (2016). Preclinical evaluation of mesenchymal stem cells overexpressing VEGF to treat critical limb ischemia. Mol. Ther. Methods Clin. Dev..

[44] Fierro F.A., Magner N., Beegle J., Dahlenburg H., Logan White J., Zhou P., Pepper K., Fury B., Coleal-Bergum D.P., Bauer G. (2019). Mesenchymal stem/stromal cells genetically engineered to produce vascular endothelial growth factor for revascularization in wound healing and ischemic conditions. Transfusion.

[45] Melly L., Cerino G., Frobert A., Cook S., Giraud M.N., Carrel T., Tevaearai Stahel H.T., Eckstein F., Rondelet B., Marsano A. (2018). Myocardial infarction stabilization by cell-based expression of controlled Vascular Endothelial Growth Factor levels. J. Cell. Mol. Med..

[46] Yeh T.S., Fang Y.H.D., Lu C.H., Chiu S.C., Yeh C.L., Yen T.C., Parfyonova Y., Hu Y.C. (2014). Baculovirus-transduced, VEGF-expressing adipose-derived stem cell sheet for the treatment of myocardium infarction. Biomaterials.

[47] Yu J.X., Huang X.F., Lv W.M., Ye C.S., Peng X.Z., Zhang H., Xiao L.B., Wang S.M. (2009). Combination of stromal-derived factor-1alpha and vascular endothelial growth factor gene-modified endothelial progenitor cells is more effective for ischemic neovascularization. J. Vasc. Surg..

[48] Park J.S., Bae S.-H., Jung S., Lee M., Choi D. (2019). Enrichment of vascular endothelial growth factor secreting mesenchymal stromal cells enhances therapeutic angiogenesis in a mouse model of hind limb ischemia. Cytotherapy.

[49] Feener E.P., King G.L. (1997). Vascular dysfunction in diabetes mellitus. Lancet.

[50] Waltenberger J. (2001). Impaired collateral vessel development in diabetes: Potential cellular mechanisms and therapeutic implications. Cardiovasc. Res..

[51] Loomans C.J., de Koning E.J., Staal F.J., Rookmaaker M.B., Verseyden C., de Boer H.C., Verhaar M.C., Braam B., Rabelink T.J., van Zonneveld A.J. (2004). Endothelial progenitor cell dysfunction: A novel concept in the pathogenesis of vascular complications of type 1 diabetes. Diabetes.

[52] Sorrentino S.A., Bahlmann F.H., Besler C., Muller M., Schulz S., Kirchhoff N., Doerries C., Horváth T., Limbourg A., Limbourg F. (2007). Oxidant stress impairs in vivo reendothelialization capacity of endothelial progenitor cells from patients with type 2 diabetes mellitus: Restoration by the peroxisome proliferator-activated receptor-gamma agonist rosiglitazone. Circulation.

[53] Yan J., Tie G., Park B., Yan Y., Nowicki P.T., Messina L.M. (2009). Recovery from hind limb ischemia is less effective in type 2 than in type 1 diabetic mice: Roles of endothelial nitric oxide synthase and endothelial progenitor cells. J. Vasc. Surg..

[54] Hayes K.L., Messina L.M., Schwartz L.M., Yan J., Burnside A.S., Witkowski S. (2018). Type 2 diabetes impairs the ability of skeletal muscle pericytes to augment postischemic neovascularization in db/db mice. Am. J. Physiol. Cell Physiol..

[55] Tepper O.M., Galiano R.D., Capla J.M., Kalka C., Gagne P.J., Jacobowitz G.R., Levine J.P., Gurtner G.C. (2002). Human endothelial progenitor cells from type II diabetics exhibit impaired proliferation, adhesion, and incorporation into vascular structures. Circulation.

[56] Hazarika S., Dokun A.O., Li Y., Popel A.S., Kontos C.D., Annex B.H. (2007). Impaired angiogenesis after hindlimb ischemia in type 2 diabetes mellitus: Differential regulation of vascular endothelial growth factor receptor 1 and soluble vascular endothelial growth factor receptor 1. Circ. Res..

[57] Emanueli C., Caporali A., Krankel N., Cristofaro B., Linthout S., Van Madeddu P. (2007). Type-2 diabetic Lepr(db/db) mice show a defective microvascular phenotype under basal conditions and an impaired response to angiogenesis gene therapy in the setting of limb ischemia. Front. Biosci..

[58] Fadini G.P., Miorin M., Facco M., Bonamico S., Baesso I., Grego F., Menegolo M., de Kreutzenberg S.V., Tiengo A., Agostini C. (2005). Circulating endothelial progenitor cells are reduced in peripheral vascular complications of type 2 diabetes mellitus. J. Am. Coll. Cardiol..

[59] Vasa M., Fichtlscherer S., Aicher A., Adler K., Urbich C., Martin H., Zeiher A.M., Dimmeler S. (2001). Number and migratory activity of circulating endothelial progenitor cells inversely correlate with risk factors for coronary artery disease. Circ. Res..

[60] Hill J.M., Zalos G., Halcox J.P., Schenke W.H., Waclawiw M.A., Quyyumi A.A., Finkel T. (2003). Circulating endothelial progenitor cells, vascular function, and cardiovascular risk. N. Engl. J. Med..

[61] Werner N., Kosiol S., Schiegl T., Ahlers P., Walenta K., Link A., Böhm M., Nickenig G. (2005). Circulating endothelial progenitor cells and cardiovascular outcomes. N. Engl. J. Med..

[62] Yan J., Tie G., Wang S., Messina K.E., DiDato S., Guo S., Messina L.M. (2012). Type 2 diabetes restricts multipotency of mesenchymal stem cells and impairs their capacity to augment postischemic neovascularization in db/db mice. J. Am. Heart Assoc..

[63] Amin A.H., Abd Elmageed Z.Y., Nair D., Partyka M.I., Kadowitz P.J., Belmadani S., Matrougui K. (2010). Modified multipotent stromal cells with epidermal growth factor restore vasculogenesis and blood flow in ischemic hind-limb of type II diabetic mice. Lab Investig..

[64] Biscetti F., Gentileschi S., Bertucci F., Servillo M., Arena V., Angelini F., Stigliano E., Bonanno G., Scambia G., Sacchetti B. (2017). The angiogenic properties of human adipose-derived stem cells (HASCs) are modulated by the High mobility group box protein 1 (HMGB1). Int. J. Cardiol..

[65] Hou Y., Ryu C.H., Jun J.A., Kim S.M., Jeong C.H., Jeun S.S. (2014). IL-8 enhances the angiogenic potential of human bone marrow mesenchymal stem cells by increasing vascular endothelial growth factor. Cell Biol. Int..

[66] Liew A., Baustian C., Thomas D., Vaughan E., Sanz-Nogués C., Creane M., Chen X., Alagesan S., Owens P., Horan J. (2018). Allogeneic Mesenchymal Stromal Cells (MSCs) are of Comparable Efficacy to Syngeneic MSCs for Therapeutic Revascularization in C57BKSdb/db Mice Despite the Induction of Alloantibody. Cell Transpl..

[67] Huang W.H., Chen H.L., Huang P.H., Yew T.L., Lin M.W., Lin S.J., Hung S.C. (2014). Hypoxic mesenchymal stem cells engraft and ameliorate limb ischaemia in allogeneic recipients. Cardiovasc. Res..

[68] Tateishi-Yuyama E., Matsubara H., Murohara T., Ikeda U., Shintani S., Masaki H., Amano K., Kishimoto Y., Yoshimoto K., Akashi H. (2002). Therapeutic angiogenesis for patients with limb ischaemia by autologous transplantation of bone-marrow cells: A pilot study and a randomised controlled trial. Lancet.

[69] Huang P., Li S., Han M., Xiao Z., Yang R., Han Z.C. (2005). Autologous transplantation of granulocyte colony-stimulating factor-mobilized peripheral blood mononuclear cells improves critical limb ischemia in diabetes. Diabetes Care.

[70] Lu D., Chen B., Liang Z., Deng W., Jiang Y., Li S., Xu J., Wu Q., Zhang Z., Xie B. (2011). Comparison of bone marrow mesenchymal stem cells with bone marrow-derived mononuclear cells for treatment of diabetic critical limb ischemia and foot ulcer: A double-blind, randomized, controlled trial. Diabetes Res. Clin. Pract..

[71] Ozturk A., Kucukardali Y., Tangi F., Erikci A., Uzun G., Bashekim C., Sen H., Terekeci H., Narin Y., Ozyurt M. (2012). Therapeutical potential of autologous peripheral blood mononuclear cell transplantation in patients with type 2 diabetic critical limb ischemia. J. Diabetes Complicat..

[72] Li M., Zhou H., Jin X., Wang M., Zhang S., Xu L. (2013). Autologous bone marrow mononuclear cells transplant in patients with critical leg ischemia: Preliminary clinical results. Exp. Clin. Transpl..

[73] Procházka V., Gumulec J., Jalůvka F., Šalounová D., Jonszta T., Czerný D., Krajča J., Urbanec R., Klement P., Martinek J. (2010). Cell therapy, a new standard in management of chronic critical limb ischemia and foot ulcer. Cell Transpl..

[74] Powell R.J., Marston W.A., Berceli S.A., Guzman R., Henry T.D., Longcore A.T., Stern T.P., Watling S., Bartel R.L. (2012). Cellular therapy with Ixmyelocel-T to treat critical limb ischemia: The randomized, double-blind, placebo-controlled RESTORE-CLI trial. Mol. Ther..

[75] Powell R.J., Comerota A.J., Berceli S.A., Guzman R., Henry T.D., Tzeng E., Velazquez O., Marston W.A., Bartel R.L., Longcore A. (2011). Interim analysis results from the RESTORE-CLI, a randomized, double-blind multicenter phase II trial comparing expanded autologous bone marrow-derived tissue repair cells and placebo in patients with critical limb ischemia. J. Vasc. Surg..

[76] Henry T.D., Schaer G.L., DeMaria A., Recker D., Remmers A.E., Goodrich J., Patel A.N. (2016). The ixCELL-DCM Trial: Rationale and Design. Cell Transpl..

[77] Patel A.N., Henry T.D., Quyyumi A.A., Schaer G.L., Anderson R.D., Toma C., East C., Remmers A.E., Goodrich J., Desai A.S. (2016). Ixmyelocel-T for patients with ischaemic heart failure: A prospective randomised double-blind trial. Lancet.

[78] Mohammadzadeh L., Samedanifard S.H., Keshavarzi A., Alimoghaddam K., Larijani B., Ghavamzadeh A., Ahmadi A.S., Shojaeifard A., Ostadali M.R., Sharifi A.M. (2013). Therapeutic outcomes of transplanting autologous granulocyte colony-stimulating factor-mobilised peripheral mononuclear cells in diabetic patients with critical limb ischaemia. Exp. Clin. Endocrinol. Diabetes.

[79] Gupta P.K., Chullikana A., Parakh R., Desai S., Das A., Gottipamula S., Krishnamurthy S., Anthony N., Pherwani A., Majumdar A.S. (2013). A double blind randomized placebo controlled phase I/II study assessing the safety and efficacy of allogeneic bone marrow derived mesenchymal stem cell in critical limb ischemia. J. Transl. Med..

[80] Bura A., Planat-Benard V., Bourin P., Silvestre J.S., Gross F., Grolleau J.L., Saint-Lebese B., Peyrafitte J.A., Fleury S., Gadelorge M. (2014). Phase I trial: The use of autologous cultured adipose-derived stroma/stem cells to treat patients with non-revascularizable critical limb ischemia. Cytotherapy.

[81] Xie B., Luo H., Zhang Y., Wang Q., Zhou C., Xu D. (2018). Autologous Stem Cell Therapy in Critical Limb Ischemia: A Meta-Analysis of Randomized Controlled Trials. Stem Cells Int..

[82] Liu Y., Xu Y., Fang F., Zhang J., Guo L., Weng Z. (2015). Therapeutic Efficacy of Stem Cell-based Therapy in Peripheral Arterial Disease: A Meta-Analysis. PLoS ONE.

[83] Wahid S.F.A., Ismail N.A., Jamaludin W.F.W., Muhamad N.A., Hamid M.K.A.A., Harunarashid H., Lai N.M. (2018). Autologous cells derived from different sources and administered using different regimens for “no-option” critical lower limb ischaemia patients. Cochrane Database Syst. Rev..

