# Supplementary material for: The Role of the Stem Cells Therapy in the Peripheral Artery Disease
Source: Int J Mol Sci. 2019 May 7;20(9):2233. doi: 10.3390/ijms20092233 (PMC6539394; doi:10.3390/ijms20092233)
Supplement: Supplementary file 1 [file ijms-20-02233-s001.pdf]

Author	Year	Patient population	Type of stem cells	Immunophenotype	Blinding	Results	Notes
**Tateishi-Yuyama et al.**	2002	CLI without surgical option	Bone-marrow derived mononuclear cells (mixed population)	Both CD34+ and CD34- cells	Complete blinding until randomization; only principal investigators unblinded after randomization. Unspecified outcome assessment	Increase in ABI, TcO2 and pain-free walking time in the treatment group. Increase in collateral vessel formation as seen at MRA	Marked improvement in TcPO2 and rest pain, with a majority of patients free from rest pain at 4 and 24 weeks. Showed that CD34- cells expressed angiogenic factors
**Tateishi-Yuyama et al.**	2002	CLI without surgical option	Bone-marrow derived mononuclear cells (mixed population)	Both CD34+ and CD34- cells	Complete blinding until randomization; only principal investigators unblinded after randomization. Unspecified outcome assessment	Increase in ABI, TcO2 and pain-free walking time in the treatment group. Increase in collateral vessel formation as seen at MRA	Marked improvement in TcPO2 and rest pain, with a majority of patients free from rest pain at 4 and 24 weeks. Showed that CD34- cells expressed angiogenic factors
**Huang P et al.**	2005	Diabetic CLI without severe coronary, cerebral or renal vascular disease	G-CSF mobilized peripheral blood mononuclear cells	Mixed population	Unclear, possibly no blinding at all		Marked improvement. No blinding, no formal sample size assessment, randomization procedure unclear
**Lu D et al.**	2011	Diabetic CLI	Bone-marrow derived mononuclear cells (mixed population) in treatment group 1 and Bone-marrow derived mesenchymal stem cells in treatment group 2	Mixed population in group 1; group 2: CD29+, CD71+, CD90+, CD105+, CD45-, CD34-	Reported double blinding, procedure of blinding not specified; single center	Both MNCs and MSCs performed better than control group. MSCs performed better than MNCs with respect to all endpoint except pain scale at rest. Ulcer healing also occurred earlier with MSCs compared to MNCs. Both treatment groups had no amputations, while 6	Higher production of angiopoietic factors by MSC than MNC

						amputations were performed in the control group	
**Ozturk A et al.**	2012	Diabetic type 2 CLI	GM-CSF mobilized peripheral blood mononuclear cells	CD34+	Unspecified	Increase in ABI, TcO2 and rest pain scale in the treatment group compared to control group. Also improved ulcer healing and amputation-free survival in treatment group compared to control group.	
**Powell RJ et al.**	2012	No surgical options CLI	Mixed population (at lease 98% of CD90+ and CD45+ cells)		Reported	Significant reduction of the composite endpoint of treatment failures in the treatment group compared to control	Multicenter study. Almost also 6% less new gangrene. Phase 2 study. Used as efficacy endpoint a composite of all treatment failures (time to treatment failures, including mortality, amputation, doubling of ulcers size, de novo gangrene)
**Prochazka V et al.**	2010	CLI with failed surgical or endovascular options	Mixed population of bone marrow stem cells, no sorting or immunophenotyping	Not performed, CD34+ cells counted before injection	Unspecified	Half amputation in the treated group compared to the control group	
**Mohammadzadeh et al.**	2013	Diabetic CLI with failed revascularization or no surgical options	Peripheral blood mononuclear cells after G-CSF mobilization	Unspecified	Unspecified		
